# AI ethics and systemic risks in finance

**DOI:** 10.1007/s43681-021-00129-1

**Published:** 2022-01-13

**Authors:** Ekaterina Svetlova

**Affiliations:** University of Twente, Enschede, The Netherlands

**Keywords:** Artificial intelligence, Systemic risks, AI ethics, Responsibility, Finance

## Abstract

The paper suggests that AI ethics should pay attention to morally relevant systemic effects of AI use. It draws the attention of ethicists and practitioners to systemic risks that have been neglected so far in professional AI-related codes of conduct, industrial standards and ethical discussions more generally. The paper uses the financial industry as an example to ask: how can AI-enhanced systemic risks be ethically accounted for? Which specific issues does AI use raise for ethics that takes systemic effects into account? The paper (1) relates the literature about *AI ethics* to the *ethics of systemic risks* to clarify the moral relevance of AI use with respect to the imposition of systemic risks, (2) proposes a theoretical framework based on *the ethics of complexity* and (3) applies this framework to *discuss implications for AI ethics* concerned with AI-enhanced systemic risks.

## Introduction

In the last decade, we have observed the widespread use of algorithms based on artificial intelligence (AI) techniques. The ethical issues related to the rise of AI are numerous. The burgeoning field of AI ethics has been concerned with aspects of AI use such as transparency, fairness, non-maleficence, privacy and accountability [1, 2]. Notably, most of those issues have been analysed at the level of individual users and algorithms: how can we ethically evaluate the negative effects of the overuse, misuse or malfunction of *particular* AI programs for *specific* groups of individuals (e.g. clients, university applicants, defendants, etc.)? However, the potential harms that require ethical consideration might stem from system-wide interactions of AI applications with each other and with humans [3]. The collective behaviour of algorithms can produce detrimental social effects, for example, in warfare [4], climate change [5], social media [6] and business (e.g. in e-commerce or supply chains). Thus, AI ethics should pay attention to morally relevant *systemic effects* of AI use.

In this paper, I outline the directions such ethics could take using the example of financial markets. In this field, AI technology has been profoundly transforming how clients are advised, investment decisions are made and trades are executed. This transformation has been speeding up and intensifying processes of computerisation and market automation that have been underway for decades. However, whereas the early-day algorithms were quite simple and merely performed, or enacted, human-defined rules (such as mean reversion or momentum) at a high speed, the more advanced contemporary algorithms apply AI techniques, such as machine-learning,^1^ to *independently discover* strategies on the basis of which they invest and trade [7, 8]. I will pay a particular attention to this qualitative difference in the course of the paper.

Importantly, the literature has already discussed at length whether applications of advanced technologies such as AI could contribute to systemic risks in financial markets [7, 9–12]. It has explored whether these technologies can potentially harm the financial system—and the economy as a whole—and thus jeopardise the life chances of citizens only marginally involved in investing and trading. Based on this discussion, there are reasons to assume that widespread AI use in finance could exacerbate such risks. Independent or light-touch-controlled AI programs—while interconnecting and interacting with human market participants and with each other—can cause negative systemic events such as a liquidity crunch, price collapse or severe market disruptions. These risks have not only economic but also ethical significance.

The moral relevance of systemic risks in finance has been recently addressed by researchers in the field of finance ethics [13–16]. They problematized the negative unintended consequences of activities in financial markets for individuals who are remote from the stock exchanges and trading rooms and hardly contribute to the build-up of systemic risks. These societal groups (which are mostly unspecified in advance) might become exposed to unemployment, poverty, home loss and deterioration of health as a result of turbulence on financial markets. This unwanted exposure raises issues of justice, fairness and responsibility. However, the unintended and indirect character of systemic risks makes the ethical assessment of such issues very challenging.

While recognising the moral relevance of systemic risk imposition in the field of finance, the literature in finance ethics has not related those risks to the rise of technology, particularly AI. However, keeping in mind the rise of AI and its potential relevance for financial stability, we have to ask: How can AI-enhanced systemic risks in financial markets be ethically accounted for? Which specific issues does AI use raise for ethics that takes systemic effects into account?

To answer these questions, the paper first identifies a lacuna between three strands of research: (1) literature on the governance of systemic risks in finance that claims that AI has the potential to contribute to systemic risks, however, *without reflecting on its ethical implications*; (2) general AI ethics that *neglects* morally relevant *systemic effects* of technology; (3) the finance ethics of systemic risks, which is not explicitly concerned with *technological advances* such as AI. This discussion allows clarification of the moral relevance of AI use with respect to the imposition of systemic risks and assessment of the difficulties faced by long-standing approaches in AI ethics in treating this moral problem. For example, the assignment of individual responsibility for potentially significant systemic events and dealing with the low moral salience of systemic harms are particularly challenging.

To address the identified challenges, I suggest a framework based on the *ethics of complexity* [17–20]. This concept was developed to address ethical issues from the system’s view and is particularly useful for analysing the imposition of systemic risks in technologically advanced financial markets. In the final step of the paper, the principles of the ethics of complexity are adopted to discuss the implications for ethics concerned with AI-enhanced systemic risks.

The paper is conceptual and normative. Its contribution is twofold. First, it draws the attention of ethicists and practitioners to a particular set of risks related to AI use, namely systemic risks. These risks have been neglected so far in AI-related professional codes of conduct, industrial standards and ethical discussions about AI more generally. Second, the paper proposes a theoretical framework that strives to overcome the current focus of AI ethics on individual responsibility, transparency and technical fixes (“design for values”). The ethics of complexity and its applications to AI ethics generate structural, social and relational perspectives on AI-related moral issues.

## AI use could contribute to the imposition of systemic risks

The use of AI in trading execution and asset management is on the rise. It is a continuation of the processes of computerisation, market automation and big data development that have been going on for decades, for example, in the hedge fund industry, high-frequency trading (HFT) or ETF firms. However, AI applications bring about some qualitative differences. In contrast to algorithms from the early days, they have the ability to develop and implement their investing and trading strategies independently of their programmers and users. In other words, AI strategies might not be based on data, rules and correlations that human investors or traders consider to be important, but can deliberate and generate new trading policies and investment rules by themselves.

For example, machine learning applications discover datasets humans would not consider relevant; for instance, a program can find out that CEOs’ tweets are more informative than annual reports, which have been the central source of information about companies until now. AI algorithms analyse social media posts, news trends and macroeconomic data presented to them in a variety of forms such as audio, pictures, maps and texts. Deep neural networks could replace human investors in analysing fundamental data provided by companies (e.g. sales, debt, profit, etc.) to recognize regularities in data (pattern recognition) and identify the most promising stocks for a portfolio and determine the size of an investment [8].^2^

Although most trades (ca. 80%[21]) are automated today, advanced AI techniques still drive a rather minor subset of trades and investments in financial markets [7]. Nevertheless, their development is accelerating and requires attention, also from the side of ethicists. This is because there are reasons to think that the wide use of AI could impose morally relevant systemic risks in financial markets.

The World Economic Forum [9], Financial Stability Board [7] and BaFin^3^ [22] as well as the growing academic literature [10–12, 72, 73] highlight the fact that AI applications might destabilize the financial system and make it more prone to crises *in principally new ways*. First, financial companies that widely apply data-driven and, thus, easily scalable business models could become new, systemically important providers. Second, systemic risks may arise when actors and their AI algorithms start following similar strategies and thus moving markets in the same direction (herding), increasing the risk of severe market disruptions. This could happen if a large number of market participants use identical or very similar AI algorithms and data sources (for example, when algorithms and data are made available by a small number of providers). Herding might also arise because technological know-how is quickly spread across markets—through transfer of employees, reverse engineering and copying of successful algorithms—propagating the use of similar tools. Third, AI applications could lead to stronger interconnectedness of human and algorithmic market participants through new types of contract and relationship [9, 72]. For example, WEF [9] envisages the possibility that AI systems autonomously learn to collude with each other. They might destabilize competition by continuously bidding against each other to achieve the highest or lowest market price for a particular stock. This behaviour could lead to “algorithmic collusion”, the situation in which algorithms learn to engage in anti-competitive behaviour, which could cause severe market swings and jeopardize other market participants and firms whose share price is affected.

More generally, financial AI applications, which are programmed to guess and outsmart each other, are based on mutual observations and are thus prone to locking in their actions, leading to herding, disastrous resonance and tail events. Hence, *the interaction order of algorithms, or collective machine behaviour,* becomes central to better understanding systemic risks in markets [23, 72].

Finally, humans do not disappear but continue to interact with technology, contributing to complexity and unintended consequences. For example, WEF [9] describes the possibility that human traders stop understanding markets characterized by excessively complex technology at a particular point in time and thus stop acting; machines interpret the lack of buyers as “bad sentiment” and continue to sell at lower prices, enhancing volatility and systemic risks. On the human side, the inability to “understand markets” due to the opaqueness of applied AI technologies might also lead to market disruptions [7, p. 26]: “If in doubt, users of […] AI and machine learning tools may simultaneously pull their ‘kill switches’, that is manually turn off systems. After such incidents, users may only turn systems on again if other users do so in coordinated fashion across the market. This could thus add to existing risks of system-wide stress”.

We received a foretaste of such developments on March 16, 2020, when investors observed the coronavirus-related mini-crash in the financial markets. All of the most important world indices dropped by 12–13%, including Dow Jones Industrial Average (DJIA) [24]. Professor Andrew Lo from MIT claimed that the fall was caused by synchronized losses among AI models: “What we saw in March of 2020 is not unlike what happened in 2007, except it was faster, it was deeper, and it was much more widespread” [25]. In the quote, he was referring to the so-called “quant” crash in August 2007, when several quantitatively managed hedge funds simultaneously suffered severe losses [26, 27].

A lively debate also emerged about the flash crash on May 6, 2010, and the mini-crash on the US Treasury market on October 15, 2014. Those events raised the question of whether market automation (through high-frequency trading algorithms in this case) caused and amplified mini-crashes and, thus, destabilized financial markets. Official reports on both mini-crashes [28, 29] as well as academic literature on the topic [30–35] showed that market complexity and opacity were enhanced by algorithms’ interactions, making it difficult to isolate the importance of individual traders and firms. The Treasury report [29, p. 33] concluded that “analysis of participant-level data in the cash and futures markets did not reveal a clear, single cause of the price movement during the event window on October 15”. Still, the report highlighted the strong interdependence between human and algorithmic market players as an issue that should be watched to better understand future market crashes. More generally, the literature on the 2010 flash crash underlined the importance of a *systemic view* on modern financial markets in which events such as mini-crashes and other systemic disruptions result from non-linear complex feedback loops and interactions between algorithms and humans [30].

All of these discussions provide reasons to assume that there is a connection between applications of advanced AI technologies and the potential rise of systemic risks.^4^ Indeed, collective machine behaviour as well as hybrid human–machine behaviour might initiate negative emergent effects at the level of financial system as a whole [3]. Such negative systemic effects can cause severe social harm and thus constitute an important aspect of AI use requiring a rigorous ethical evaluation.

## AI ethics neglects the systemic effects of new technologies

One would expect to find outsets of such an evaluation in the burgeoning field of AI ethics. However, this strand of research has widely neglected the systemic effects of AI applications so far. As the recent reviews of the relevant academic literature [2] and surveys of ethical guidelines developed in various industries [1, 36, 37] demonstrate, AI ethics has been concerned with ethical principles that are primarily of a technical nature and relate to the activities of individual algorithms and users. This also applies to the discussions about AI in business and finance ethics [38, 39].

The concerns raised so far have been related to the following questions: How do we ensure that the decisions driven by a particular AI algorithm are fair and do not re-produce accidental biases? How do we guarantee the privacy of data if an algorithm malfunctions? Given the very nature of AI (i.e. its ability to learn and decide autonomously), how can designers and users of AI be held accountable if they know little about the consequences of AI actions and cannot control them [38, 40]? In particular, doubts have been raised as to whether the traditional ideals of *transparency* [41] and *explainability* [9], as pre-conditions for holding someone accountable, can be easily fulfilled in the case of AI algorithms. All these core concerns related to AI ethics strongly focus on technical aspects of individual actions and decisions involving AI: which negative and direct effects might the overuse, misuse or malfunction of AI have for particular groups of individuals (such as clients and users)? How to ensure *accountability in the form of transparency*? To what extent is it possible to guarantee privacy or fairness through the design and technical fixes of an algorithm?

Systemic risks fall between the cracks of this debate because they are of a different nature. They build up even if no one misuses the technology and no algorithm malfunctions. They arise from collective but non-intended actions and potentially cause harm to persons who are neither originators nor addressees of those actions. The moral relevance of the imposition of such risks has been widely neglected in ethical debates about AI. For example, systemic effects are not a part of the detailed maps of AI-related ethical concerns provided by Mittelstadt et al. [42] and Whittlestone et al. [2]. While analysing a number of AI ethics guidelines and reports, Hagendorff [1, p. 103] observed:

[…] almost no guideline talks about AI in contexts of care, nurture, help, welfare, social responsibility or ecological networks. In AI ethics, technical artefacts are primarily seen as isolated entities that can be optimized by experts so as to find technical solutions for technical problems. What is often lacking is a consideration of the wider contexts and the comprehensible relationship networks in which technical systems are embedded.

Thus, discussions about AI ethics should be complemented by a structural or complex perspective that takes into consideration “how technology shapes the broader environment in ways that could be disruptive or harmful” [43].

The importance of taking a systemic perspective in ethics has, however, not remained completely unnoticed. Business ethicists and political economists addressed this issue in the aftermath of the Great Financial Crisis in 2008 but did not pay sufficient attention to AI and other advanced technologies.

## The ethics of systemic risks neglects technology

The financial crisis of 2008 was a paramount example of a systemic event that drew the attention of ethicists in the fields of business and finance ethics [7, 9–12] and the political economy [45, 46] to systemic risks. The merit of this excellent research was to, first, clearly argue the case of the moral relevance of systemic risks and, second, highlight the challenges that existing ethical approaches face, while analysing moral aspects of systemic risk imposition.

Most cases of systemic risk deserve ethicists’ attention because they involve a specific asymmetry between the originators and the potential victims (or moral patients). What is morally problematic is not the eventual harm as such, but the building up of risks that are *imposed* on vulnerable members of society.

[T]he group of agents contributing most to the generation of systemic risks are usually not identical to the group of agents most detrimentally affected by the materialisation of risk. Materialisation of systemic risk can involve systemic breakdown or impairment, with the effects being not only largely indiscriminate, but often disproportionately affecting the most vulnerable with the least means to protect themselves. This is not the same as absolute losses incurred, but rather refers to reduced ability to lead a secure, healthy and fulfilling existence and to make choices that improve one’s material existence. [46, p. 5].

For example, a crash on the stock markets in New York and London might affect the income or employment situation of a Spanish farmer who has never actively invested in equities himself [45].

Research on the ethics of systemic risks argues that the central impediment to analysing systemic risk imposition lies in the difficulty in isolating the extent to which an individual action contributes towards the joint consequences. In the complex, often not fully understood, cause-and-effect chains of catastrophic events, the contribution of an individual action remains unclear and marginal (the problem of unstructured, collective harm [15]). In other words, there is no clearly demonstrable, knowable and quantifiable relationship between the actions of individual actors and the consequences of their actions. This is often referred to as a *problem of many hands* in philosophy [47] and *the tragedy of commons* in economics [48].

Systemic risks might result from the actions of *morally prudent* agents who are unaware of the detrimental outcomes of their actions at the system level. James [45, p. 253] terms this phenomenon*’innocence' borne of uncertainty* and states:

[…] as long as (1) moral principles are essentially for self-governance (and so subject to justification according to what addressed agents can do, what they can know, and the expected outcomes of their actions, etc.), and (2) the agents in question are not all-knowing and omnipotent, but limited in their epistemic and other agential powers, then it is possible, in principle, for such agents to suffer terrible outcomes in which no one is morally at fault.

Floridi [49] coined the term “faultless responsibility”: the evil state of the world is brought about through local interactions that are not as such morally loaded but neutral.

Such situations are the blind spot of many existing ethical approaches (such as deontological ethics, consequentialist ethics, stakeholder theory, integrative social contracts theory and plural subject theory) [13, 15, 16, 49] because most of the theories are based on the deliberation of clear connections between agents and the outcomes of their actions. The connections are usually established through *intentionality* (agents’ intent to achieve an outcome), *knowledge* (agents know how they contribute to an outcome) and *control* (agents can control an outcome). Ethics—and the assignment of responsibility—without these three conditions is challenging.

The central point that the ethics of systemic risks makes is that marginal, only indirectly traceable and non-intentional contributions are not fully free from responsibility. Still, this individual responsibility is very difficult to assign: the suggestions in the literature range from “no one is at fault” [13, 14] to “everyone is responsible” [49]. The other problem is that the originators of systemic risks not only do not know whether they actually contribute to the build-up of future catastrophic events but also are unaware of who (potentially) suffers as a result of such events and when. Hence, they are not motivated to think carefully about the moral implications of their actions: the moral salience of systemic harms is quite low [16].

For the purpose of this paper, it is important to highlight that this theoretical debate about the ethics of systemic risks had not been related to the spread of new technologies such as computer modelling practices, algorithms and market automation. Generally, technology has seldom been an issue in ethical debates about finance [50]. Admittedly, some work has been done on the ethics of high-frequency trading [51–53]. However, this research is primarily concerned with *purposeful manipulation strategies* such as spoofing, layering, wash sales or quote stuffing. In the case of systemic risks, however, morally problematic outcomes might come about without malicious behaviour by human or algorithmic agents.

Thus, keeping in mind the fact that AI algorithms might enhance detrimental systemic effects in financial markets (see Sect. 2), the important work on the ethics of systemic risks presented above should be extended to include considerations about the advances of new technologies. We have to ask: how to account for morally relevant systemic risks that build up with AI participation? What difference does the use of AI make for ethical considerations of systemic risks?

## AI exacerbates the challenges for established ethical concepts

The first observation that I would like to make is that the use of AI enhances the difficulties ethicists face in analysing systemic risks. The challenges of intentionality, knowledge and control become more pronounced due to the complexity of individual AI algorithms and the complexity of their interplay [54]. There is not only the problem of *many hands* but also the problem of *many things* [54], meaning that ethicists need to account for AI technologies that interact with each other and human agents. Because AI activities have an element of creativity and autonomy and often surpass human cognitive capacity, they install additional layers of complexity between actions and their (also collective) outcomes. What AI algorithms of the new generation do cannot be exhaustively described by codes or programs; rather, there are hidden and complex mid layers of statistically trained algorithmic elements which are difficult or impossible to fully understand due to their complexity.^5^ On top of this, the limited understanding of how AI algorithms interact with each other and with human participants makes markets themselves a highly complex black box with rapidly shifting interlinkages and risks. It is difficult to anticipate how AI-based algorithms will act collectively due to feedback loops,^6^ emerging (self-organizing) properties and tight couplings [72]. Thus, AI programmers and users might be unsure about the causal contribution of their actions to the negative collective final outcome which is the very subject of moral evaluation. The “breach” of culpable causation between individual decisions and joint consequences (as already highlighted in the previous section) widens the “responsibility gap” in the case of AI-induced systemic risks.

Moreover, unawareness of the moral relevance of one’s actions, or *moral ignorance*, becomes particularly pronounced with the widespread use of AI in finance. As the literature already suggests, reliance on complex mathematical models and algorithms in finance causes moral distancing between users of technology, on the one hand, and the affected people and places, on the other hand [50, 55]. West [55, p. 602] observes that, in financial markets, “as the level of complexity increases, the considerations of moral obligations tend to decline”. Technology mediates the relation to others and increases detachment from moral obligations [56]. “Epistemic invisibility” becomes “moral invisibility” [57, p. 287]. Thus, moral distancing and detachment, which are anyway a significant challenge for the ethics of systemic risks, are even more eminent in the case of AI-induced systemic risks, i.e. when the affected people are remote from the markets, unknown to AI designers and users and the relation to them is mediated by a complex technology.

Thus, AI ethics that accounts for systemic risks has to address the epistemic, control- and motivation-related challenges described above. We are in need of an ethical approach that adopts the systemic view (on financial markets), puts interactions and relations between human and algorithmic agents at the core and takes the limited knowledge and uncertainty about systemic outcomes and individual contributions to these outcomes seriously. I suggest utilising the *ethics of complexity* as a pathway towards such a concept.

## The ethics of complexity as a framework for AI ethics

*The ethics of complexity* [17–20] directly addresses the challenges encountered by moral reasoning in situations that require systems thinking. AI-enhanced systemic risks provide a point in case for such ethics. The ethics of complexity understands detrimental systemic events as *emergent* events that result from the interactions of actants and are formed here and now. As a result, each actant (for example, an AI programmer in an investment company) is ignorant of the exact mechanism that guides the behaviour of the system as a whole and thus cannot know the full effects of his or her actions. He or she responds to the information that is locally and provisionally available in his or her web of relationships and interactions. The problem is further exacerbated by the fact that systemic risks might result from the *collective behaviour of algorithms* which is exceptionally difficult to anticipate.

While highlighting the ever-incomplete knowledge about complex systems, the ethics of complexity departs from *ethical intellectualism* [58], which presupposes the foreseeability of harm and the knowledge of causes and effects as a basis for ethical judgments, and is at the core of most ethical concepts. The relevance of an intentional action and the ability to control systemic events are also questioned.

### On the limitations of the moral obligation to know

Let us discuss the epistemic condition in more details. The established ethical approaches have considered the limits of knowledge at the level of agents as a *culpable* but principally *surmountable* obstacle on the way to the ideals of accountability, traceability, transparency and explainability. For instance, in an analysis of the causes of the financial crisis in 2008, De Bruin [13] points to the limited knowledge about complex financial products of customers, banks, credit rating agencies and regulators. They apparently lacked the competence to properly inquire into the major characteristics of the products and form beliefs about them. According to De Bruin, the solution from an ethical point of view would be to acquire and exercise epistemic virtues, for example love of knowledge, the courage to assess evidence and revise beliefs, justice with respect to opposing positions and humility to search and accept a wide range of opinions. Those virtues would “enlarge the likelihood of gaining knowledge” [13, p. 70] and enable people not to miss, but to access and process relevant information (p. 42). In other words, and applied to AI, *expanding knowledge* about technology is a moral virtue which the involved actors should acquire and develop. Based on these considerations, while discussing systemic risks, Moggia [15] argues for the *ethics of knowledge acquisition*: *the agent is responsible for the process of knowledge acquisition itself*. This concept is generally in line with ethical arguments about technological innovations. As innovations always produce ignorance and uncertainty, actors should pursue a prudent strategy of reducing uncertainty and acquiring knowledge [59, 60].

Although the ethics of complexity does not deny the *moral obligation to know*, it always starts with the unattainability of full knowledge about relevant complexities. This unattainability is a basic feature of contemporary electronic, highly automated, partly AI-driven markets. For example, the efforts to develop explainable artificial intelligence (XAI) are important but may remain ever incomplete due to the very nature of AI. So, in the case of financial markets, AI will develop trading programs other than those expected and understood by its designers, and these programs will interact with each other in ways that remain opaque to the latter. As a result, actions in situations of *not knowing* become an important issue for AI ethics: how to define ethical behaviour under conditions of radical uncertainty and ignorance? [61] This question might become central to AI ethics inspired by the ethics of complexity.

Thus, to assign moral responsibility, ethicists should be concerned with manifold types of *imperfect knowledge*. They should seek to identify situations in which ignorance is culpable or excusable and why. Agents who are ignorant of the consequences of their actions can be found to be blameworthy not because they do not know but because they could—and should—have known. At the same time, the *difficulties* and limitations in acquiring relevant knowledge in complex situations should be taken into account. An agent might be concerned about a relevant issue (e.g. the contribution of his or her algorithm to systemic risks) but be faced with insuperable obstacles in meeting his or her epistemic duties (due to the genuinely untransparent nature of a deep neural network algorithm, for example, or the limited understanding of collective machine behaviour). To hold someone responsible, we must assume that those difficulties *can* be overcome. However, “under certain epistemic circumstances, one could remain ignorant despite having inquired extensively, and without having been reckless or negligent in the management of one’s morally relevant beliefs” [62, p. 49].

As discussed above, there are many factors that impede efforts to know about individual AI contributions to systemic risks. The central question is therefore what an individual AI user should—and could—*reasonably* know [63]. If we find out that an agent is expected to acquire knowledge that is unavailable, the agent’s ignorance is epistemically non-culpable. The relevant ethical question is then whether we can formulate any *reasonable obligations* that govern the formation of an agent’s belief. In other words, ethics in the era of AI has to realize that agents have to ethically deal with unknowns, and those unknowns should be ethically judged. These insights from the ethics of complexity could give the debate on the accountability and transparency of AI a new twist which I will discuss below.

### The ethics of complexity as relational ethics

The other important aspect of the ethics of complexity is that it takes seriously the already discussed difficulties in assigning *individual responsibility* for systemic events. While acknowledging these difficulties, most approaches in AI ethics as well as ethical codes developed by companies and across industries unisono claim that responsibility for all consequences of AI use nevertheless lies with the individual designers or users (e.g. [38]). But if we recognize that systemic events result from the *interactions* of system’s elements, then it might make sense to consider an ethical framework that tries to “focus not on individual components but on their relationships” [19, p. 941]. This thinking, which is central to the ethics of complexity, has already started to feed into AI ethics.

First, while addressing the difficulties in assigning responsibility in the web of interactions of *many hands* and *many things*, Coeckelbergh [54, p. 2058, original emphasis] claims that “[…] it is important to clarify all […] structural and temporal relations and interactions: not only social interactions and roles of humans but also their interactions with things and relations and interactions *between* things”. On that basis, he proposes developing a *relational* approach to the responsibility problem: “Responsibility is not only about doing something and knowing what you are doing; it also means *answerability*” (p. 2061) towards those who are affected by the use of AI (moral patients). This concept echoes *the patient-oriented approach* suggested by Floridi [49].

Second, in a similar vein, Ananny [41, p. 98] claims that algorithmic ethics is genuinely relational: “It matters little if the black boxes of algorithm code […] are opened or comprehensible since they only become ethically significant in relation to others”. He defines AI algorithms “as an assemblage of institutionally situated computational code, human practices, and normative logics that creates, sustains, and signifies relationships among people and data through minimally observable, semiautonomous action” (p. 99). This relational understanding of algorithms upstages the concerns about transparency (“reading black boxes”) and emphasizes ethical evaluations of such assemblages, i.e. the possibilities they open, the power they provide and the unexpected consequences they produce (“looking across algorithms”). Such a relational approach might be helpful for *AI ethics concerned with systemic effects*.

In sum, the ethics of complexity establishes a number of original points of departure which help to address the difficulties that standard ethical approaches have with respect to AI and systemic risks. In the remainder of the paper, I will discuss in more detail how conceptual insights of the *ethics of complexity* could be adopted in AI ethics for the analysis of AI-enhanced systemic effects.

## Implications for AI ethics

### Focus on morally relevant systemic effects

First of all, the ethics of complexity draws attention to the morally relevant, system-level *outcomes* of non-linear, non-deterministic interactions between humans and machines as well as between algorithms. It means that moral patients, for example, people outside the financial system whose life chances and rights might be jeopardized by the potentially detrimental joint outcome of human and machine interactions in financial markets, become the focal point of ethical considerations. AI ethicists should clarify the morally unacceptable consequences of imposing systemic risks on those and other relevant groups whose rights to fairness, freedom and justice might be infringed. They should also investigate whether the intensity and scope of potential harm varies across the groups of moral patients at the systems level. The necessity to take a broader systemic view and include the societal effects of AI in the list of relevant ethical issues applies to AI ethics in all fields, not only in finance: “[E]thics codes must adopt both an agency *and* structural approach to encompass a wide range of AI risks” [44, p. 4, my emphasis]. Increasing awareness of the importance of the systemic effects of AI would be an important achievement of AI ethics informed by the ethics of complexity.

### Ethics and the regulation of systemic risks in automated markets

However, in finance, we often encounter the argument that systemic risks have been taken care of by regulators, making ethical considerations about those risks redundant. Can the law replace ethics? Undeniably, systemic risks have become the focus of regulatory concern in the financial sector in the last decade (e.g. Basel III, MiFID II, the German Banking Act and Regulation Systems Compliance and Integrity adopted by the U.S. Securities and Exchange Commission (SEC)). At the same time, all of these regulatory initiatives place a legal responsibility on *agents* who design and apply algorithms in investing and trading. Regulators often follow a reductionist approach and assume that there is an unambiguous, linear causal mechanism according to which an individual component in the system (e.g. an investment firm) causes an unwanted outcome at the macro level (e.g. financial market instability). For example, watchdogs require investment firms to comply with given order limits to prevent erroneous orders which may contribute to a disorderly market. They also stipulate that these firms continuously test and monitor their algorithms, have circuit breakers in place which allow them to immediately pause trading in an emergency situation and regularly provide a self-assessment and validation report on their risk mitigation measures [73]. Some countries, e.g. Germany, have introduced an algorithm-tagging rule which requires firms to identify which algorithm is used to generate a trading decision with a number [66]. Furthermore, oversight bodies focus on identifying individual and intentional manipulative behaviour such as spoofing. In other words, company-internal risk management systems and processes are considered to be a solution for the system-wide problem (systemic risk).

Although undeniably, all those measures are important for maintaining financial stability, they do not comply with the ethics of complexity. The complexity view on markets suggests, first, that systems failures arise from the relationships between agents and not (only) from the malfunctioning of individual agents. Second, harm is not necessarily caused by negligent or manipulative actions, but also by ‘neutral’ actions. Third, regulatory measures at the individual level which aim to prevent the propagation of systemic risks might—maybe paradoxically—enhance such risks and weaken the stability of markets. For example, Min and Borch [74, p. 18] argue that “stop-loss orders [as a measure for preventing markets’ failure] may trigger a downward spiral when interacting with other stop-loss orders”. The same applies to the circuit breakers prescribed by law. When the majority of market participants withdraw from trading, the market might be destabilized. Finally, in contrast to regulation, while dealing with complex phenomena such as systemic risks, the ethics of complexity assumes that knowledge about those phenomena and, in particular, about the causal mechanisms behind them is inevitably provisional, local and generated in an exploratory process. As a result, it is difficult (or even impossible) to formulate unambiguous measures for preventing systemic risks.

Clearly, ethics and the law are not necessarily aligned. The law that regulates automated activities in financial markets must formulate legal requirements for *individual* market participants and firms, even if it starts with the (moral) concern about possible negative market outcomes. Ethics—as the systematic evaluation of morally relevant issues—“is both more and less than the law: it is more because many ethical concerns are not addressed by the law and less because the outcome of ethical considerations is not necessarily transformed into legal norms” [75, p. 297] at the level of individual behaviour. Thus, whereas the law cannot adopt a uniquely systemic view and inevitably has to shift its focus to the level of individual activities, ethics can continue to be concerned with the evaluation of morally relevant outcomes at the systems level.

More generally, ethics can provide a wider framework which is not necessarily forced into the straightjacket of rules which are often outdated, nation-bound and circumvented by their addressees [15, 16]. That is why the challenges systemic risks pose in banking and finance cannot be overcome by regulation alone and why ethics is a crucial component for dealing with them. Thus, in the next step, we have to contemplate how the ethics of complexity can complement regulation and which actionable solutions it offers for the burgeoning field of AI ethics in finance.

### Focus on relationships and the role of intermediaries

The ethics of complexity might suggest focusing on the *relations* within the system—the relations between human and algorithmic market participants as well as those between AI algorithms. Based on the discussion about the pathways to systemic risks in AI-enhanced financial markets in Sect. 2, we can claim that those relationships are primarily responsible for systemic risks and not individual, isolated actions. For example, a risk of procyclical behaviour might be enhanced when trading AIs copy each other’s models and data sources. Hence, the *relationships* between human and algorithmic market participants should be the fundamental unit of ethical analysis.

This conceptual move might help to connect AI ethics to contemporary debates on complexity in finance, macro-prudential regulation and economics [76, 77]. Many *relational* concepts such as networks, feedback loops and interaction effects are at the core of the modern finance discourse about systemic risks and financial stability and could provide inroads for considering the ethics of complexity in this context.

Besides academic research, the establishment of such a connection would require the clarification of concrete steps and actionable solutions. One important step might be to introduce an ethical intermediary which would be concerned with AI ethics and would adopt the framework of ethics of complexity, as discussed in the paper at hand. The role of such an intermediary could be played, for example, by *professional associations* in the financial industry, as discussed by Herzog ([16], p. 532): She envisaged them to be “a focal point of professional ethics, education and moral debate”. At the same time, WEF [9, p. 74] suggested to assign a similar role to *system-wide intelligence hubs.* They could closely cooperate with financial institutions and serve, for example, as think tanks, best practice repositories and arbiters in times of crashes. In contrast to oversight bodies, such institutions would not have to follow a reductionist approach. They could maintain the ethical focus on system-level issues such as morally relevant relations, networks, feedback loops and tight coupling within the system.

With respect to *what* these intermediaries can do and *how* they do it, three central aspects might be worth considering: they can (1) identify relevant relationships and ethically evaluate them, (2) collect information and knowledge that provide a basis for determining the *moral obligations to know* from the systemic perspective and (3) ensure consultation of multi-stakeholders. Let us discuss all three activities in the above order.

#### Ethical evaluation of relevant relationships

An ethical intermediary should identify which relationships are crucial for the emergence of systemic risks and to what extent. For example, is the relation between an AI designer and AI algorithm as important as the relation between a trader and an algorithm? A particular focus should be on the machine-machine relationships: which AI programs tend to copy each other, collude with each other and herd particularly strongly? Can this question be answered? How exactly do the ways in which algorithms interact in markets increase the probability of the unwanted outcomes at the systems level?

Furthermore, the central issue in the case of AI-enhanced systemic risks, as discussed in Sect. 5, is the “broken” and distanced relation between AI firms and the potential victims of systemic risk imposition. There is no direct way of “repairing” this relationship, for example, through answerability in the form of communication, as suggested by Coeckelbergh [54], because a direct dialogue between the two parties is impossible. The ethics of complexity would, however, suggest shaping those relations in a way that allows the moral distance between AI users and those who might experience harm from AI use to be overcome. Thus, the accountability relation can be *enhanced* by introducing an intermediary that substitutes or represents accountability holders who are not able to directly exercise control over those who are supposed to be constrained by the accountability mechanism [64].

#### Defining obligations to know

As discussed, until now, most ethical and regulatory approaches have argued for *obligations to know* at the level of individual AI designers, traders or firms; it is their moral responsibility to make an effort to understand or find out the impact of their actions on the morally relevant outcome, e.g. the systemic risks [13, 14, 20]. However, the ethics of complexity claims that the production of relevant knowledge cannot be solely a task of individual market participants. Knowledge about channels through which AI enhances systemic risks cannot be produced at the level of companies. Keeping in mind the inevitable incompleteness and local character of agents’ knowledge about the workings of the complex financial system, it is unrealistic that individual AI designers and users fully know the interconnections of their algorithms and, as a result, unrealistic that they are able to estimate their (potential) contribution to systemic risks.

Thus, the moral obligation to know should remain at the systems level; e.g. it should be the responsibility of the suggested ethical intermediaries. Intermediaries could provide *an infrastructure* for developing and exchanging relevant knowledge [16] about how AIs interplay in markets and influence them. For example, the new ways in which AI might contribute to systemic risks discussed in Sect. 2 are hypotheses that need close observation and checking based on data. This would be an important task for the ethical intermediaries in question.

So far, efforts to understand the emergence of systemic risks in automated complex markets have been insufficient. To date they have focused on high-frequency trading (HFT) which is qualitatively different from the effect of machine-learning algorithms. First, the discussion on HFT has primarily been about speed (e.g. [32]), not about the algos’ ability to develop strategies independently from humans, to cooperate with each other and directly copy each other’s strategies, etc. Second, the efforts have been backward-looking and occasional, often after a detrimental event, for example the investigations into the flash crashes of 2010 and 2015 by CFTS/SEC and the Treasury discussed above. Third, those investigations and other similar efforts have demonstrated that it is difficult to acquire relevant knowledge about the emergence of systemic risks, not only for individual AI designers and users but even for regulators who have better access to information about market transactions [66, 67]. Despite the introduction of algorithm-tagging rules, account identifiers and so forth, “we are … still far from having a robust understanding of how trading algorithms interact” [23, p. 55]. Hence, many more non-reductionist epistemic efforts are needed in this respect. This crucial *moral obligation to know* can be fulfilled by an ethical intermediary.

Such epistemic efforts can partly take the form of “landscape assessment”, which is discussed in [65] as an important pre-condition for making the principles of AI ethics actionable:

Promising areas for landscape assessment include the technical state-of-the art, identifying which capabilities and applications exist, which societal, economic and political factors may affect their potential proliferation or market penetration to various actors, and the resulting timelines of sociotechnical change; such assessments also include the societal environment, to determine what are the public’s and policymaker’s overall understanding, range of concerns and ability to engage with issues. Finally, it could serve to review the legislative status quo, to understand the scope of issues already covered. [65, p. 15]

Importantly, such a landscape assessment would help to circumvent the common criticism that the principles of AI ethics do not make use of adequate knowledge and, as a result, often lack any connection to technological, legal and social reality.

At the same time, the ethics of complexity would advocate reflection on the limitations of epistemic efforts and the consideration of morally relevant *non-knowledge*. In this respect, an important task of an ethical intermediary would be to investigate what is not known now, what cannot be known in principle and will most likely never be known. For example, it might be considered whether it is at all possible to identify the contribution to systemic risks of an individual AI or a single company that applies AI. A clear description of zones of uncertainty and areas where knowledge is *not possible* could be a crucial part of landscape assessment and allow designation of areas of human and algorithmic fallibility. This way of thinking would relativise the transparency ideal and the ultimate emphasis on *efforts to know* an algorithm or a program. Instead of blame-shifting, it is important to provide a productive environment for understanding market events and responses to them similar to other safety–critical industries (e.g. aerospace) [9]. Indeed, collecting and sharing *knowledge about non-knowledge* and defining *reasonable obligations to know* might become a central task of an intermediary in the field of ethics concerned with AI and its systemic effects.

#### Consultations with multiple stakeholders

Essentially, recent studies in AI ethics [38, 41] suggest that the knowledge relevant for discussions about AI is genuinely social and relational. Considering algorithmic accountability [38], claim that knowing does not simply come from looking into codes, but from understanding the assemblage of human and non-human actors. As a result, *morally relevant knowledge* might be produced not by an individual or a company, but by a network that includes members of various professional groups and their technologies. It is thus the task of the ethical intermediaries in question to organize and coordinate such a network.

At the current fledgling stage of AI ethics in business and finance, it is crucial that ethical intermediaries start consolidating the opinions of different professional groups [16] *before* formulating ethical codes and principles. In the same vein, while discussing the ethics of high-frequency trading, Davis et al. [52] argue not to focus solely on traders but to include “quants”, software engineers and computer specialists. Aligning their conflicting perspectives might provide a foundation for a new *cross-disciplinary* ethical standard that aims “[t]o reduce the likelihood of a breakdown in the global trading mechanism” (p. 871) and to prevent detrimental outcomes, including potential harm caused by the imposition of systemic risks. More generally, Stix [65] suggests that multi-stakeholder consultations and cross-sectional feedback are central for the development of *actionable* AI ethical principles.

These considerations might also apply to financial markets in the era of AI. An ethical intermediary under discussion should cooperate with already established regulatory and oversight bodies that are concerned with systemic risks in financial markets and have already produced a remarkable amount of knowledge on the topic (e.g. Bank for International Settlement and Financial Stability Board). Furthermore, the agency that mediates between AI-driven markets and moral patients could also collect the views and ethical concerns from both sides: companies that apply AI and their employees, on the one hand, and citizens and the civic public, on the other hand. Insights from such consultations could help to strengthen the accountability relation and be later incorporated into codes and standards.

Finally, the ethics of complexity suggest that ethical intermediaries or hubs use multi-stakeholder consultations and cross-sectional feedback to clarify the relevant core values and tensions between them [2, 36]. For example, the meaning of “fairness” in the case of AI-enhanced systemic risks might be different from the case of a biased individual AI. In the first situation, “fairness is first and foremost a distributional question” [46, p. 13]. In other words, it is a question of distribution of risks; it would be unfair if individuals who do not participate in markets and display low or reasonable risk-taking behaviour were subjected to detrimental life-changing effects resulting from the interplay of many unknown algorithmic and human agents (and the eventual materialization of systemic risk). In the case of a biased AI algorithm, fairness is an issue when some members of a *clearly defined group* are affected and suffer disadvantages resulting from the decisions of *a particular algorithm*. In other words, the systemic view propagated by the ethics of complexity requires the elucidation of ethical values which are never universal but contextual and temporary. According to ([19], p. 944), it is “ethical to aim for diversity [of opinions]” because the “diversity of narratives can be seen as an enormous source of resilience in complex systems”. To maintain this diversity could be an important task for ethical intermediaries.

## Conclusion

In sum, the discussion in the paper demonstrates that AI ethics that considers the systemic effects of AI is still in the infant state. The paper proposes conceptually advancing AI ethics using the framework of ethics of complexity. It shows that this move would allow AI ethics to focus on morally relevant system-level issues. The proposed framework understands ethics as genuinely relational and considers human–machine or machine-machine relationships as units of analysis. This shift of the ethical focus from non-intended, often morally neutral actions of human and algorithmic agents to ethical issues at the systems level and relationships between agents is particularly suitable for ethics that take into account collective machine behaviour, that is, the interaction order of algorithmic agents that do not possess intentionality in the common sense, are genuinely opaque, etc. Advancing this framework is a difficult yet important task for future ethical research on AI.

Next to severe theoretical challenges, AI ethics that is concerned with systemic risks also faces some practical impediments. Before AI ethicists will be able to develop and apply their instruments—such as professional codes and standards of business behaviour based on ethical values—many preliminary steps should be taken: the awareness for moral relevance of systemic risks should be raised, the relevant professional groups defined, the exchange of views between these groups facilitated, the meaning of values and tensions between these values clarified and obligations to know should be defined, including limits to these obligations.

Such tasks cannot be fulfilled by individual AI firms or their employees and should be delegated to an intermediary, an ethical authority at the systems level. Such an institution might be an important interim step in the development of AI ethics concerned with systemic risks and would be able to inform and consult practitioners on ethical matters.

## Footnotes

1 Generally, AI can be defined as „a system’s ability to interpret external data correctly, to learn from such data, and to use those learnings to achieve specific goals and tasks through flexible adaptation “ [68, p. 17]. Machine-learning is a sub-field of AI and „may be defined as a method of designing a sequence of actions to solve a problem, known as algorithms, which optimise automatically through experience and with limited or no human intervention “ [7, p. 4].

2 These are only a few examples. For a detailed discussion of AI applications in investments and trading see [7, 69, 70]. They specify the meaning, possibilities and limitations of supervised learning, unsupervised learning, reinforced learning as well as Artificial Neural Networks and deep learning in finance.

3 German Federal Institute for Financial Services Supervision.

4 For a more general argument beyond finance see [71].

5 Referred in the literature as the “black box” problem.

6 *Interactions* between AI programs affect the involved *individual* AI programs because the latter learn and adjust their strategies while observing those interactions.
